# Fermentation Characteristics, Nutrient Content, and Microbial Population of *Silphium perfoliatum* L. Silage Produced with Different Lactic Acid Bacteria Additives

**DOI:** 10.3390/ani15131955

**Published:** 2025-07-02

**Authors:** Yitong Jin, Bao Yuan, Fuhou Li, Jiarui Du, Meng Yu, Hongyu Tang, Lixia Zhang, Peng Wang

**Affiliations:** 1 College of Animal Sciences, Jilin University, Changchun 130062, China; ytjin23@mails.jlu.edu.cn (Y.J.); yuan_bao@jlu.edu.cn (B.Y.); lifh@jlu.edu.cn (F.L.); dujr23@mails.jlu.edu.cn (J.D.); yumeng22@mails.jlu.edu.cn (M.Y.); tanghy@jlu.edu.cn (H.T.); 2 Animal Husbandry and Veterinary Bureau of Dingxi City, Dingxi 743000, China; adxmj2012@163.com

**Keywords:** silage, Fodder storage, bacterial community, fermentation quality, lactic acid bacteria additives, *Silphium perfoliatum* L.

## Abstract

The aim of this study was to explore the effects of different lactic acid bacteria additives (*Lactiplantibacillus plantarum* or *Lentilactobacillus buchneri*) on the fermentation quality, chemical composition, in vitro digestibility, bacterial community structure, and predictive function of *S. perfoliatum* silage feed. Fresh *S. perfoliatum* was wilted overnight, then its moisture content was adjusted between 65 and 70%. The experiment was performed in three groups as follows: (1) the control group (CK group), which lacked a *Lactobacillus* preparation; (2) the *Lactiplantibacillus plantarum* (*L. plantarum*) group (LP group), which was inoculated with *L. plantarum* at 5 × 10^6^ cfu/g FW; and (3) the *Lentilactobacillus buchneri* (*L. buchneri*) group (LB group), which was inoculated with *L. buchneri* at 5 × 10^6^ cfu/g FW. The results showed that *L. plantarum* significantly reduced pH and increased lactic acid (LA) content in *S. perfoliatum* silage compared with the control. *L. buchneri*, on the other hand, excelled in reducing ammonia nitrogen (NH_3_-N) content and significantly increased acetic acid (AA) content. At 60 days of fermentation, the CP content was significantly higher (*p* < 0.05) in the LP and LB groups than in the CK group (19.29 vs. 15.53 and 15.87). At 60 days of fermentation, the *iv*CPD was significantly higher (*p* < 0.05) in the LB group than in the CK and LP groups (57.80 vs. 54.77 and 55.77). The 60-day silage process completely altered the bacterial community of *S. perfoliatum* silage. In the fresh samples, the dominant genera were Weissella_A and Pantoea_A. Weissella_A and Pantoea_A were gradually replaced by *Lentilactobacillus* and *Lactiplantibacillus* after *S. perfoliatum* ensiling. After 45 days of fermentation, *L. buchneri* became the dominant strain in CK, LP and LB groups. Inoculation with *L. plantarum* altered the succession of the bacterial community from 7 to 15 days of fermentation of *S. perfoliatum*. In contrast, inoculation with *L. buchneri* affected the succession of the bacterial community from 30 to 60 days of *S. perfoliatum* fermentation. In *S. perfoliatum* silage aged 7 to 60 days, the amino acid metabolic pathway in the LB group remained upregulated. The experimental results revealed that inoculation with *L. buchneri* had a stronger effect on *S. perfoliatum* silage than inoculation with *L. plantarum*. Thus, *L. buchneri* should be selected as an additive for *S. perfoliatum* silage fermentation in practical production.

## Simple Summary

In this study, we investigated the effects of different lactic acid bacteria additives on the fermentation quality, chemical composition, in vitro digestibility, bacterial community structure, and predictive function of *Silphium perfoliatum* L. silage feed. The experimental results revealed that inoculation with *L. buchneri* had a stronger effect on improving the aerobic stability of *S. perfoliatum* silage as well as on the composition of the microbial community compared with *L. plantarum*. Our research provides the theoretical basis and support for the development of *S. perfoliatum* as a high-quality silage feed ingredient for animal nutrition.

## 1. Introduction

The current ruminant production is transferring from traditional grazing to intensive production [1]. Therefore, the feed supply in ruminant production is facing huge pressure [2] and scientists are looking for alternative feed ingredients to fulfil feed demand for ruminant production [3]. More attention has been paid to developing unconventional roughage resources in the livestock industry.

*Silphium perfoliatum* L. *(S. perfoliatum)* is a perennial herbaceous plant of the Asteraceae family that is native to North America and one of the alternative feed resources in animal feed [4]. It has a well-developed root system that can be used to prevent soil erosion and is also used as feed for pigs, cattle, sheep, and other livestock [5]. *S. perfoliatum* is extremely tolerant to temperature because it can survive and grow at temperatures ranging from −40 °C to 40 °C [6]. In addition, *S. perfoliatum* has a high yield of biomass during harvest and the biomass far exceeds that of traditional forages such as alfalfa. *S. perfoliatum* is rich in superoxide dismutase (SOD) and flavonoids, and its protein content can reach 18%, with the highest being 23% [7]. *S. perfoliatum* is also rich in amino acids, lysine, calcium, phosphorus and various vitamins, which lead to favourable palatability for livestock and poultry [8]. However, the supply of *S. perfoliatum* cannot meet a whole year’s demand due to its seasonal growth. Ensiling is an important measure for forage storage around the world [9] because ensiling can effectively preserve the protein, vitamins, and minerals of *S. perfoliatum*, and it is more easily digested and absorbed by animals after fermentation. Ensiling technology not only solves the problem associated with the seasonal supply of *S. perfoliatum*, but also significantly improves the nutritional value of feed and animal performance [10].

A large number of studies show that the addition of lactic acid bacteria (LAB) improves storage and feed flavour [11,12,13,14]. Homofermentative and heterofermentative LAB are widely used inoculants in silage production. Therefore, we hypothesise that inoculating LAB in *S. perfoliatum* silage may have valuable insights on improving fermentation quality. *Lactiplantibacillus plantarum* is a homozygous fermenting LAB that exhibits rapid growth and multiplication [15]. Compared with other strains of LAB, it has high acid resistance. *L. plantarum* has a clear competitive advantage when competing with other bacteria for fermentation substrates [16]. *L. plantarum* uses the substrate to produce lactic acid (LA) in the early stages of fermentation, thus rapidly creating an acidic environment and minimising nutrient loss [17,18]. The pH and ammonia nitrogen content were significantly lower in the *L. plantarum* group compared to the control without the LAB preparation. The result was that LA and acetic acid (AA) contents were significantly increased, thus improving the fermentation quality of silage [19]. As a heterogeneous fermenting LAB, *Lentilactobacillus buchneri* (*L. buchneri*) exhibits unique metabolic properties during fermentation. This strain is able to convert soluble carbohydrates into AA and 1,2-propanediol [20]. This metabolic pathway contributes to the aerobic stability of silage, thus prolonging its preservation time. Using high-throughput sequencing technology, we found that microbial additives could alter the structure of microbial communities, thereby improving the fermentation quality of silage [21,22]. However, there is little information on the effect of *L. plantarum* or *L. buchneri* on microbial communities present in *S. perfoliatum* silage.

In order to gain insight into the effect of different inoculants on silage, *L. plantarum* and *L. buchneri* were selected in this study to systematically evaluate their effects on the fermentation quality, chemical composition, in vitro digestibility, and aerobic stability of *S. perfoliatum* silage. The effects of LAB additive types and ensiling time on the composition and dynamics of bacterial communities during the ensiling process were also explored.

## 2. Materials and Methods

### 2.1. Silage Preparation

*S. perfoliatum* seeds were purchased from Jiyuan HeYi Agricultural Science and Technology Ltd., Co. The seeds were sown on 23 May 2024 and the plants were harvested on 28 September 2024 at the standard forage trial site in Tongyu County, Baicheng city, Jilin Province, China (45°13′ N, 123°25′ E, and 300 m above sea level). *S. perfoliatum* were wilted overnight (approximately 21 h) to bring the moisture content to between 65% and 70%, after which the *S. perfoliatum* plants were cut into 2 to 3 cm pieces using a chopper (FTT-2500C, Dongguan Fangtai Electrical Co., Dongguan, China). The *S. perfoliatum* material (500 g) was sealed in 185 mm × 210 mm polyethylene silage bags using a vacuum plastisol sealing machine (EV-60, Nissami Packaging Machinery Co., Beijing, China). The LAB strains used in this experiment included *L. plantarum* LP1 and *L. buchneri* 9-2. *L. plantarum* LP1 was purchased from Snow Seal Seedling Co. (Sapporo, Japan), and *L. buchneri* 9-2 was purchased from Gansu Puno Beikang Biotechnology Co. (Lanzhou, China). The experiment was divided into the following three treatments: (1) control group (CK group), which lacked a *Lactobacillus* preparation; (2) *L. plantarum* LP1 group (LP group), which inoculated with *L. plantarum* at 5 × 10^6^ cfu/g FW; and (3) *L. buchneri* 9-2 group (LB group), which was inoculated with *L. buchneri* at 5 × 10^6^ cfu/g FW. LAB were suspended in distilled water according to the provided instructions and then sprayed evenly on grass using a small sprayer. A total of 72 silage silos (3 additives × 5 silage days × 4 replicates + 3 additives × 4 replicates) were prepared in this experiment and randomly divided into the above three treatments (CK group, LP group, LB group). Sixty silos were used to analyse the fermentation quality, chemical composition, in vitro digestibility, and microbial. The remaining 12 silos were used for aerobic stability testing after 60 days of anaerobic fermentation. All samples were homogenised before subsampling; silage samples were sampled in four replicates at the time of opening, and after sampling, the samples were stored at −80 °C.

### 2.2. Fermentation Quality

Firstly, 180 mL of distilled water was added to 20 g of silage sample and stirred with a glass rod to ensure that the sample was well mixed with the water. This was stored at 4 °C overnight. The mixture was then filtered through quantitative filter paper [23]. pH was determined with a pH metre (PH-2701, Mingrui Electronic Technology Co., Ltd., Guangzhou, China). In addition, NH_3_-N content was determined via water vapour distillation [24]. The contents of AA, LA, butyric acid (BA), and propionic acid (PA) were determined by Agilent HPLC 1260 (column: USHD Hilic-Amide, Hengspu Sheng Scientific Instruments Co., Shenzhen, China; temperature: 50 °C; flow rate: 1 mL/min; SPD: 210 nm).

### 2.3. Chemical Composition

In this experiment, chemical composition and fermentation quality were measured non-repeatedly (different samples at each time point). Silage samples and fresh raw materials were placed into a blast drying oven (101-0B, Li-Chen Bangxi Instrument Technology Co., Ltd., Shanghai, China) at 60 °C for 72 h until the samples reached a constant weight. Air-dried feed samples were obtained after 24 h of moisture return. When moisture in the material is dried, it absorbs moisture from the air, which may affect the final measurement data. Therefore, the sample must be left for 24 h before testing to allow the moisture content in the sample to reach equilibrium. The dried samples were crushed with a pulveriser (WK-150A, Qingzhou Maidusen Pharmaceutical Machinery Factory, Weifang City, China) and passed through a 1 mm sieve, followed by storage in self-sealing bags. The dry matter (DM) content was determined via the drying method at 60 °C, and the organic matter (OM) content was determined by a muffle furnace (BOS-4-10, Boshi Testing Equipment Co., Ltd., Xiamen, China). The crude protein (CP) content was determined by the Kjeldahl method (ATN-1100, Hongji Instrument and Equipment Co., Ltd., Shanghai, China). Neutral detergent fibre (NDF), acid detergent fibre (ADF), and acid detergent lignin (ADL) contents were determined using a fibre analyser (YT-SF20, Blue Rainbow Optoelectronics Technology Co., Ltd., Weifang, China) following the method of Van Soest et al. [25]. Water soluble carbohydrate (WSC) content was determined by the anthrone-sulfuric acid colorimetric method, and the buffering capacity (BC) was assayed using the method of Playne et al. [26].

### 2.4. In Vitro Digestibility

The animal welfare ethical licence number for this experiment was SY20209600, and the Chinese Guidelines for the Ethical Review of Laboratory Animal Welfare were strictly followed during the experiment. A total of 0.5 g of the dried sample was put into a 42 mm × 53 mm filter bag (Zhengfang Xingda Science and Technology Development Co., Ltd., Beijing, China). Then, they were sealed with a plastic sealer and placed into 130 mL in vitro digestive culture tubes for sealing and preservation. Six F1 generation crossbred sheep of German meat Merino and Lesser-Tailed Frosted, equipped with permanent fistulas, were selected as donors of rumen fluid (used individually for replicates). The rumen fluid was collected 1 h before the morning feeding, filtered through four layers of gauze, and placed in a 39 °C thermos flask with continuous CO_2_ ventilation. The mixed buffer solution (Na_2_HPO_4_ 9.3 g/L, MgCl_2_ 0.06 g/L, CaCl_2_ 0.04 g/L, CH_4_N_2_O 1.00 g/L, KCl 0.57 g/L, NaHCO_3_ 9.8 g/L, NaCl 0.47 g/L) was prepared according to the method of Longland et al. [27]. To prepare the mixed culture solution, the mixed buffer was mixed with rumen fluid at a ratio of 1:4. Each tube was filled with 50 mL of the culture mixture and shaken in a water bath at 39 °C for 48 h. After shaking was completed, the filter bag was rinsed and removed from the culture bottle using cold distilled water to remove any residual culture solution from the surface. The filter bag was placed in an oven and dried at 100 °C for 24 h. After drying was complete, the residues in the filter bags were weighed. These residues were used to analyse in vitro dry matter digestibility (*iv*DMD), in vitro organic matter digestibility (*iv*OMD), in vitro crude protein digestibility (*iv*CPD), and in vitro neutral detergent fibre digestibility (*iv*NDFD) in order to assess the digestive performance of the samples [28].

### 2.5. Aerobic Stability Analysis

Aerobic stability represents the resistance of silage to aerobic spoilage, and it is defined as the period required for the difference between the core temperature of the feed and the ambient temperature to reach 2 °C after the feed has been opened and exposed to an aerobic environment [29]. After 60 days of fermentation, the aerobic stability of the samples from the CK, LP, and LB groups was determined. Each sample was mixed well and transferred to a clean and sterilised 1 L plastic bucket, which was stored at ambient temperature (20.5 ± 1.5 °C). A thermocouple probe was inserted vertically into the centre of the silage sample using a multiplex automatic temperature recorder (HY-R4000, Hongyi Automation Instrumentation Co., Ltd., Changzhou, China). The sample temperature was recorded every 2 h and measured continuously for 7 days under aerobic conditions.

### 2.6. SMRT Analysis of Microbial Composition

Ten grams of fresh silage was transferred to a conical flask containing 90 mL of sterile saline and placed on a shaker for 15 min (25 °C, 120 r/min). Afterwards, it was filtered through 4 layers of gauze (30 × 40 cm, Nanchang Aokang Medical Equipment Co., Ltd., Nanchang, China) and centrifuged at 12,000× *g* rpm for 10 min to collect the slurry. The total genomic DNA of the bacteria on the surface of the silage samples was extracted using a DNA isolation kit (DP302-02; Beijing Tiangen Biochemical Technology Co., Ltd., Beijing, China). The DNA was quantified by a Nanodrop instrument, and the DNA extraction quality was checked using 1.2% agarose gel electrophoresis. DNA samples, including samples from fresh material and the silage from the CK, LP, and LB groups, were sequenced on days 7, 15, 30, 45, and 60. The conserved region of the 16S rRNA gene was amplified using universal primers (27F, 5′-AGAGTTTGATCMTGGCTCAG-3′; and 1492R, 5′-ACCTTGTTACGACTT-3′), which cover the full length of the V1–V9 region. The DNA was purified and recovered using a DNA purification and recovery kit (DP214-02, Beijing Tiangen Biochemical Technology Co., Ltd.). A full-length amplicon analysis of the microbial community’s DNA was implemented via the Pacific Biosciences Sequel III single-molecule real-time sequencing system (SMRT technology, Pacific Biosciences of California, Inc., Menlo Park, CA, USA). The downloaded bam files were exported using CCS v 4.0.0 (Generate Highly Accurate Single-Molecule Consensus Reads v4.0.0, Pacific Biosciences of California, Inc., Menlo Park, CA, USA) software to output a file format that complied with the FASTQ specification. Sequence preprocessing was performed by custom Perl scripts. Firstly, sample isolation and tag excision were performed on the basis of the molecular tag (Barcode), followed by complementary strand orientation correction by primer positioning. The QIIME2 (2019.4) software was used for analysis; sequence denoising (denoise) was performed using the DADA2 method for the quality filtering, depriming, and chimerisation steps [30]. Sequence data were subjected to quality control and were used to construct species taxonomic pedigrees with reference to the NCBI RefSeq 16S rRNA database. The phylogenetic classification of each OTU representative sequence was finally determined, and each sample was analysed for its bacterial community composition and diversity. Correlation analysis would then be performed between the top 5 bacterial genera in terms of relative abundance in the samples and LA, AA, DM, CP, NDF, ADF, NH_3_-N, *iv*DMD, *iv*CPD, and *iv*NDFD.

### 2.7. Data Analysis

General linear analyses were performed using the SPSS software (version 26, IBM, Inc., Armonk, NY, USA). Factor one was days of fermentation and factor two was additives. The interaction between the days of fermentation and additive treatments was analysed. Comparisons between groups were made using Tukey’s multiple range test, and differences were considered statistically significant at *p* < 0.05.

## 3. Results

### 3.1. Fermentation Quality of Silphium perfoliatum L. Silage

As shown in the tables (Table 1 and Table 2), from 15 to 60 days of fermentation, the LA content of the LP group was significantly higher than that of the LB group (*p* < 0.05). At 60 days of fermentation, the pH values of the LB and CK groups were significantly higher compared to the LP group (*p* < 0.05). At 60 days of fermentation, the AA content of the LP and CK groups was significantly lower than that of the LB group (*p* < 0.05). At 15 days of fermentation, the NH_3_-N content was significantly lower in the LP and LB groups compared with the CK group (*p* < 0.05). From 15 to 60 days of fermentation, the LA content was significantly higher in the LP group compared with the LB group.

### 3.2. Chemical Composition of Silphium perfoliatum L. Silage

The chemical composition of *S. perfoliatum* before and after ensiling is shown in Table 1 and Table 3. At 15 and 30 days of fermentation, the DM content was significantly higher (*p* < 0.05) in the LB group than in the CK and LP groups. At 7 to 60 days, the OM content in the CK group increased but then decreased (*p* < 0.05). In the CK group, the CP content was significantly lower (*p* < 0.05) at 60 days than at 7 days. In the LB group, the CP content increased but then decreased (*p* < 0.05). At 60 days of fermentation, the CP content was significantly higher (*p* < 0.05) in the LP and LB groups compared with the CK group. At 15, 45 and 60 days of fermentation, the NDF content was significantly lower (*p* < 0.05) in the LB group than in the CK and LP groups. At 30 to 60 days of fermentation, the ADF content was significantly lower (*p* < 0.05) in the LB group than in the CK and LP groups. As the silage period progressed, the ADL content decreased in the CK, LP, and LB groups (*p* < 0.05).

### 3.3. In Vitro Digestibility and Aerobic Stability of Silphium perfoliatum L. Silage

The in vitro digestibility of *S. perfoliatum* silage is shown in Table 4. The *iv*DMD increased but then decreased with increasing fermentation time. At 15 and 60 days of fermentation, the *iv*OMD was significantly (*p* < 0.05) lower in the CK and LP groups compared with the LB group. At 45 and 60 days of fermentation, the *iv*CPD was significantly (*p* < 0.05) lower in the CK and LP groups compared to the LB group. At 15, 45 and 60 days, the *iv*NDFD of the silage was significantly lower in the LB group than in the CK and LP groups (*p* < 0.05). Aerobic stability was significantly higher in the LB group than in the control group, and significantly lower in the LP group than in the control group.

### 3.4. Bacterial Community Diversity of Silphium perfoliatum L. Silage

**Figure 1:**
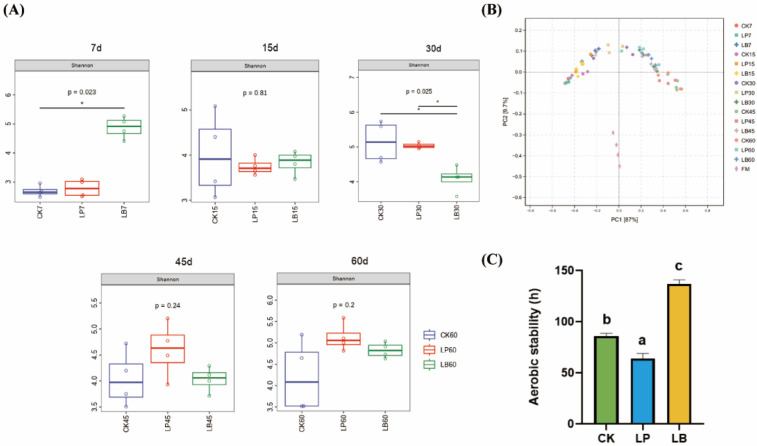
Dynamic succession of the bacterial community of *Silphium perfoliatum* L. CK, control group (no additive group); LP, *Lactiplantibacillus plantarum* added group; LB, *Lentilactobacillus buchneri* added group. (**A**) Shannon index of communities. * *p* ≤ 0.05. (**B**) PCoA was used to calculate the community differences between groups and fermentation times. (**C**) Aerobic stability of CK, LP, and LB groups. ^a–c^ Means of the additive treatments with significant differences between superscript letters (*p* < 0.05).

The Shannon index for the *S. perfoliatum* silage is shown in Figure 1A. The indices tended to increase but then decreased in each group. At 7 days of silage, the Shannon index was significantly higher in the LB group than in the CK and LP groups (*p* < 0.05). At 30 days of silage, the Shannon index was significantly higher in the LB and LP groups than in the CK group (*p* < 0.05). The bacterial community structure of the *S. perfoliatum* silage was evaluated using a principal coordinate analysis with Bray–Curtis dissimilarity for different fermentation periods and additive treatment groups. As shown in Figure 1B, the bacterial communities in the CK, LP and LB groups were clearly separated from those in Fresh weight (FM) by axis 1. There was no significant separation of the bacterial communities between the silage samples from each group at 7, 15, 30, 45 and 60 days.

### 3.5. Bacterial Community Composition and Succession of Silphium perfoliatum L. Silage

**Figure 2:**
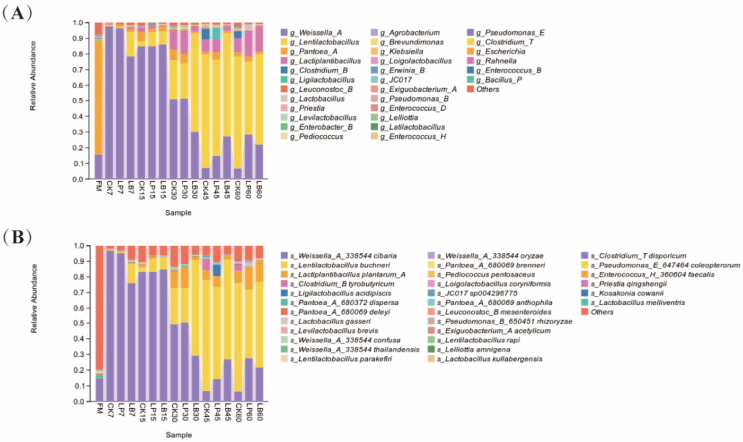
Composition and succession of the bacterial community of *Silphium perfoliatum* L. during silage. CK, control group (no additive group); LP, *Lactiplantibacillus plantarum* added group; LB, *Lentilactobacillus buchneri* added group. The number of days of silage is indicated by Arabic numerals 7, 15, 30, 45, and 60. (**A**) Relative abundance at the genus level of *Silphium perfoliatum* L. bacteria for different groups and fermentation times. (**B**) Relative abundance at the bacterial species level of *Silphium perfoliatum* L. in different groups and fermentation times.

**Figure 3:**
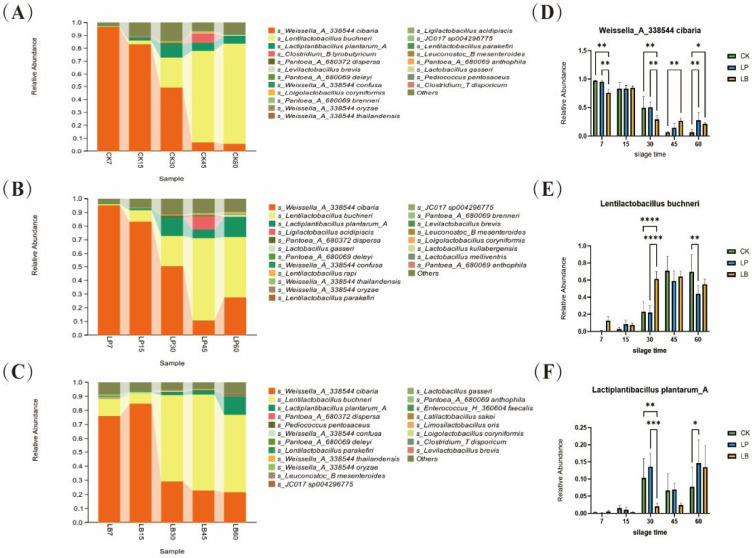
Bacterial community composition and succession in different groups of *Silphium perfoliatum* L. during silage. CK, control group (no additive group); LP, *Lactiplantibacillus plantarum* added group; LB, *Lentilactobacillus buchneri* added group. The number of days of silage is indicated by Arabic numerals 7, 15, 30, 45, and 60. (**A**) Relative abundance of bacterial species during silage in the CK group; (**B**) Relative abundance of bacterial species during silage in the LP group; (**C**) Relative abundance of bacterial species during silage in the LB group. (**D**–**F**) Relative abundance of TOP3 bacterial species of *Silphium perfoliatum* L. silage. * *p* ≤ 0.05; ** *p* ≤ 0.01; *** *p* ≤ 0.001; **** *p* ≤ 0.0001.

The bacterial community compositions of the *S. perfoliatum* silage at the genus and species levels are shown in Figure 2A,B. The bacterial community composition at the species level for each treatment group is shown in Figure 3A–C. The relative abundance of the top 3 species at the species level is shown in Figure 3D,E. At the genus level, the dominant genera in FM were Weissella_A and Pantoea_A, which were gradually replaced by Lentilactobacillus and Lactiplantibacillus after ensiling. After 7 days of fermentation, the relative abundance of Weissella_A increased to more than 78% in each group, and the relative abundance of Pantoea_A decreased to less than 1%. At 15 days of fermentation, the relative abundance of Weissella_A was between 84 and 86% in all the groups. At 30 days of fermentation, the relative abundance of Lactiplantibacillus began to increase, and the relative abundance of Weissella_A decreased. At 45 days of fermentation, Lentilactobacillus became the dominant genus, with its relative abundance increasing to more than 61%, and the relative abundance of Weissella_A ranging from 7 to 28% among the groups. At 60 days of fermentation, the relative abundance of Lactiplantibacillus increased. The addition of *Lactobacillus* significantly affected the bacterial community composition of the *S. perfoliatum* forage at the genus level. At 7 days of fermentation, the relative abundance of Lentilactobacillus was higher in the LB group than in the CK and LP groups. At the species level, the dominant strain in FM was *Weissella_A_338544 cibaria*, followed by *Pantoea_A_680372 dispersa*. After fermentation, the dominant strains in the silage were *Weissella_A_338544 cibaria*, *L. buchneri*, and *L. plantarum_A*. At 7 days of fermentation, the relative abundance of *Weissella_A_338544 cibaria* increased. At 15 days of fermentation, the relative abundance of *Weissella_A_338544 cibaria* decreased to 83%. At 30 days of fermentation, the relative abundances of *L. buchneri* and *L. plantarum_A* increased. At 45 days of fermentation, the relative abundance of *L. buchneri* continued to increase, and it became the dominant strain. The addition of *Lactobacillus* significantly affected the bacterial community composition in the *S. perfoliatum* silage at the species level. At 7 days of fermentation, the relative abundance of *L. buchneri* was higher in the LB group than in the CK and LP groups, whereas the relative abundance of *Weissella_A_338544 cibaria* was lower in the LB group than in the CK and LP groups. At 15 days of fermentation, the relative abundance of *L. buchneri* was lower in the CK group than in the LP and LB groups. At 30 days of fermentation, the relative abundance of *L. buchneri* in the LB group was significantly higher than that in the CK and LP groups, and the relative abundances of *L. plantarum_A* and *Weissella_A_338544 cibaria* were significantly lower than those in the CK and LP groups. At 45 days of fermentation, the relative abundance of *Weissella_A_338544 cibaria* was significantly higher in the LB group than in the control group. The relative abundance of *Clostridium_B tyrobutyricum* was 7.0% in the CK group, and the relative abundance of *Ligilactobacillus acidipiscis* was 7.2% in the LP group. At 60 days of fermentation, the relative abundance of *Weissella_A_338544 cibaria* was higher in the LP and LB groups than in the CK group.

**Figure 4:**
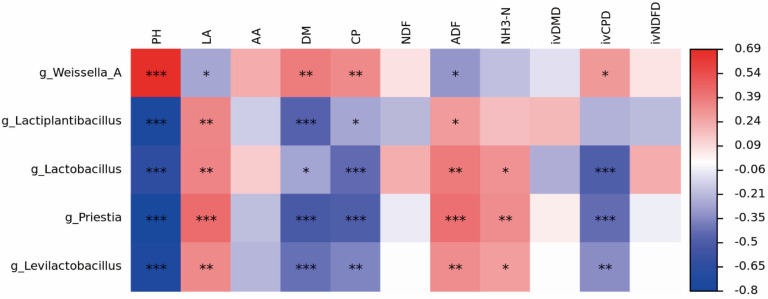
Spearman correlation heatmap of bacterial genera of *Silphium perfoliatum* L. at 7, 15, 30, 45, and 60 days silage with fermentation parameters. The colour of the squares indicates the correlation, with red being a positive correlation and blue being a negative correlation. * *p* ≤ 0.05; ** *p* ≤ 0.01; *** *p* ≤ 0.001.

The heatmap analysis of bacteria vs. indicators at the genus level for *S. perfoliatum* silage is shown in Figure 4. The pH was significantly negatively correlated with the abundances of Lactiplantibacillus, Lactobacillus, Priestia and Levilactobacillus (*p* < 0.001) but significantly positively correlated with the abundance of Weissella_A (*p* < 0.001). The LA content was significantly positively correlated with the abundance of Priestia (*p* < 0.001) but significantly negatively correlated with the abundance of Weissella_A (*p* < 0.05). The DM content was significantly negatively correlated with the abundances of Lactiplantibacillus, Priestia and Levilactobacillus (*p* < 0.001) but positively correlated with the abundance of Weissella_A (*p* < 0.05). The CP content was significantly negatively correlated with the abundances of Lactobacillus and Priestia (*p* < 0.001) but significantly positively correlated with the abundance of Weissella_A (*p* < 0.05). The ADF content was significantly positively correlated with the abundance of Priestia (*p* < 0.001) but significantly negatively correlated with the abundance of Weissella_A (*p* < 0.05). The NH_3_-N content was significantly positively correlated with the abundances of Priestia, Lactobacillus, and Levilactobacillus (*p* < 0.05). The *iv*CPD was significantly negatively correlated with the abundances of Lactobacillus and Priestia (*p* < 0.001) but significantly positively correlated with the abundance of Weissella_A (*p* < 0.001).

**Figure 5:**
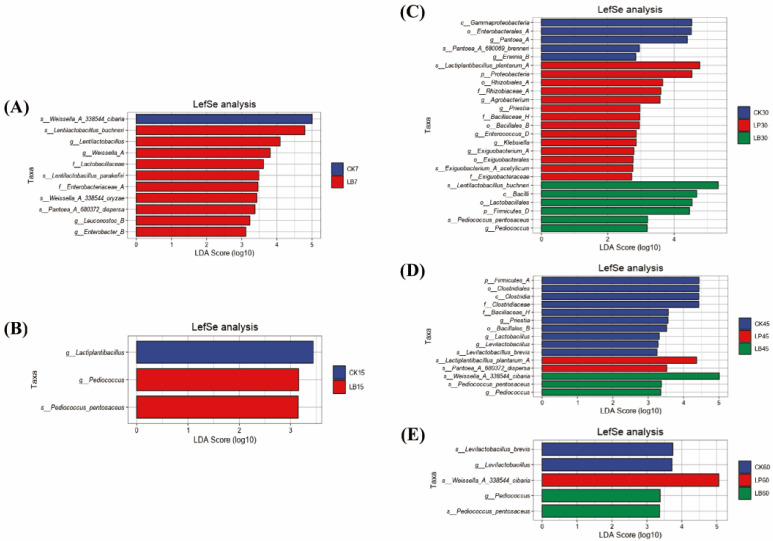
Differential bacteria identified in the three silage groups of *Silphium perfoliatum* L. (*p* < 0.05, Linear Discriminant Analysis [LDA] score > 2). (**A**) Differential bacteria identified in 7 days silage. (**B**) Differential bacteria identified in 15 days silage. (**C**) Differential bacteria identified in 30 days silage. (**D**) Differential bacteria identified in 45 days silage. (**E**) Differential bacteria identified in 60 days silage.

The results of the LEfSe analysis are shown in Figure 5A–E, which indicate that the bacterial communities differed between the treatments. At mid to late silage (15–60 days), Pediococcus in the LB group had the greatest effect on intergroup differences. At 30 days of silage, Pantoea was differentially identified in the CK group. In LP group, *L. plantarum_A*, Priestia and Klebsiella were differentially abundant bacteria. At 45 days of silage, *L. plantarum_A* was differentially identified in the LP group. Lactobacillus and Levilactobacillus were differentially identified in the CK group. At 60 days of silage, Levilactobacillus had the greatest effect on intergroup differences in the CK group.

### 3.6. Functional Predictions of Silphium perfoliatum L. Silage

**Figure 6:**
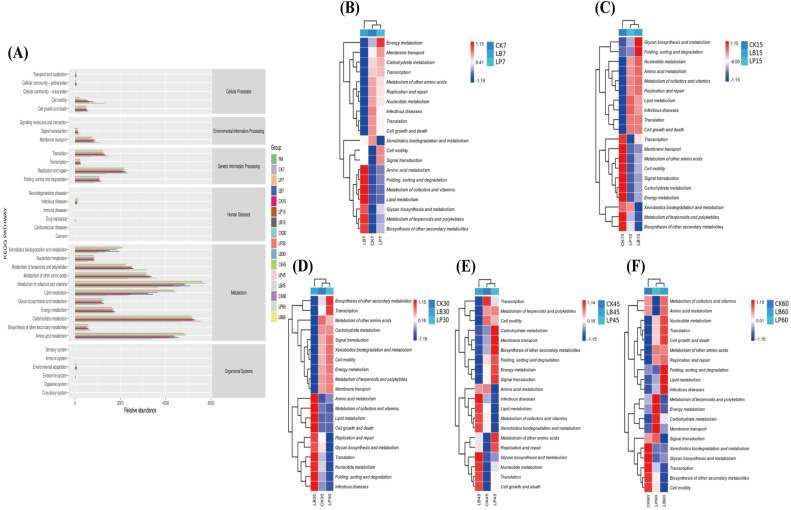
(**A**) Functional prediction of bacterial communities between different groups of *Silphium perfoliatum* L. during silage. CK, control group (no additive group); LP, *Lactiplantibacillus plantarum* added group; LB, *Lentilactobacillus buchneri* added group. The number of days of silage is indicated by Arabic numerals 7, 15, 30, 45, and 60. Functional potential prediction based on KEGG database using PICRUSt2 software. (**B**) Functional prediction of the ensiled for 7 days; (**C**) Functional prediction of the ensiled for 15 days; (**D**) Functional prediction of the ensiled for 30 days; (**E**) Functional prediction of the ensiled for 45 days; (**F**) Functional prediction of the ensiled for 60 days.

The potential function of the bacterial community of *S. perfoliatum* during different fermentation periods was predicted by the PICRUSt2 software (version 2.2.2-b, https://github.com/picrust/picrust2/wiki/, accessed on 30 December 2024) (Figure 6). Predictive functions explained by KEGG pathways were classified at the first level into cellular processes (5 pathways), environmental information processing (3 pathways), genetic information processing (4 pathways), human diseases (6 pathways), metabolism (11 pathways), and organismal systems (6 pathways). Carbohydrate metabolism and energy metabolism were significantly upregulated in the LP group compared to the CK and LB groups. Nucleotide metabolism was significantly enhanced in the LB group compared to the LP and CK groups. At the same time, amino acid metabolism as well as cofactor and vitamin metabolism were also significantly upregulated in the LB group.

## 4. Discussion

### 4.1. Effect of Different Types of LAB on the Fermentation Quality of Silphium perfoliatum L. Silage

The addition of *L. plantarum* and *L. buchneri* significantly altered the fermentation characteristics of *S. perfoliatum* silage. Compared with the control, the addition of *L. plantarum* significantly reduced the pH of *S. perfoliatum*, which was attributed to the rapid acid production by *L. plantarum* through homofermentation, directly reducing the pH and inhibiting the proliferation of spoilage bacteria. However, this phenomenon was not observed in the LB group. Kung et al. reported no significant decrease in pH when *L. buchneri* was added to alfalfa silage, and Lara et al. also observed this phenomenon in corn silage [31]. In all the groups in the present study, the LA/AA ratio was greater than 3.0, which indicated that LA fermentation had occurred [32]. The addition of *L. plantarum* increased the LA content, while the addition of *L. buchneri* increased the AA content. Bai also reported this phenomenon in corn silage [33]. AA produced by *L. buchneri* disrupts the cell membrane integrity of spoilage bacteria (e.g., Clostridium and Bacillus), inhibits their protease activity, and reduces proteolysis. The NH_3_-N content was significantly lower in the LB group than in the CK group. This was caused by the inhibition of proteolytic bacteria by *L. buchneri* via the production of AA by heterotrophic fermentation and through substrate competition and alteration of the bacterial community, resulting in a reduction in the NH_3_-N content. Heterotrophic fermentation by *L. buchneri* consumes WSCs, forcing putrefactive bacteria to start breaking down structural carbohydrates, thereby indirectly reducing the energy supply needed for deamination [34]. LA accumulation not only directly reduces the pH but also reduces the conversion of LA to AA or ethanol by inhibiting non-LAB microorganisms (e.g., Weissella). Lactiplantibacillus and Levilactobacillus showed a significant negative correlation with pH (*p* < 0.001), whereas Weissella showed a positive correlation with pH, suggesting that the first two genera play key roles in the acidification process. Furthermore, the positive correlation of Priestia with LA and *iv*CPD revealed that it may promote protein conservation through metabolites.

### 4.2. Effect of Different Types of LAB on the Chemical Composition of Silphium perfoliatum L. Silage

The addition of both *L. plantarum* and *L. buchneri* accelerated organic matter decomposition, whereas the CK group presented an early increase and then a late decrease in the OM content due to spoilage bacterial activity. High-nutritional-value silage feed also has a high CP content [35]. Compared with the CK group, the CP content in the LP and LB groups was significantly increased. This indicates that the addition of LAB can increase the CP content. Cao et al. found in their study on alfalfa silage that the application of LAB additives to alfalfa silage feed promoted CP accumulation and increased LA and AA content [36]. The CK group had a significantly lower CP content at 60 days than at 7 days of fermentation (*p* < 0.05) due to proteolysis induced by spoilage bacteria (e.g., Clostridium_B and Enterobacteriaceae). These florae degrade proteins to NH_3_-N through proteases, resulting in a decrease in the CP content. This finding is consistent with the higher NH_3_-N content in the CK group (15 to 60 days of fermentation). The CP content in the LB group increased microbial protein synthesis in the early stage of ensiling (7 to 15 days), which increased the CP content, whereas the CP content decreased in later stages (30 to 60 days) due to substrate depletion. Hou et al. found in alfalfa silage that the soluble protein content continued to rise in the seven days prior to ensiling, while NH_3_-N content increased significantly [37]. This indicates that microorganisms reintegrated free amino acids into bacterial proteins. Additionally, crude protein (CP) retention improved, and neutral detergent fibre (NDF) content significantly decreased (dry matter basis: 41.6% to 37.6%), indirectly reflecting that microbial activity enhanced nitrogen source utilisation efficiency. The addition of *L. plantarum* did not significantly affect the NDF, ADF, or ADL contents in these experiments. Importantly, the effects of LAB additives on the chemical composition of silage were not always significant [38]. As the silage period progressed, the ADL content decreased in the CK, LP, and LB groups. Traditional thinking is that ADL is quite stable and hard to break down during silage, because microbes and enzymes cannot really access it [39]. But in this experiment, the ADL content went down during the silage process of *S. perfoliatum*. The reason may be that under the unique acidic environment and complex microbial community (species with ADL degradation potential) of silage, partial degradation or structural modification of ADL occurred. At the same time, the acidic environment caused the hydrolysis of hemicellulose, making some lignin fragments soluble and thus removed in subsequent analyses. The direct degradation by fungi is also an important possible factor [40]. We will subsequently perform metagenomic or metatranscriptomic sequencing on the Artemisia annua silage samples, focusing on whether fungi (such as white rot fungi) or bacteria associated with ADL degradation are present, and whether these genes are expressed.

### 4.3. Effect of Different Types of LAB on the In Vitro Digestibility and Aerobic Stability of Silphium perfoliatum L. Silage

The *iv*DMD reflects the extent to which feed is degraded by rumen microorganisms in the rumen. An increase in the *iv*DMD is conducive to an increase in feed intake [41]. The *iv*OMD reflects the level of nutrient absorption in feed by animals and determines their production performance [42].

Compared with the CK and LP groups, the LB group had lower *iv*NDFD. Broderick et al. found that the *iv*DMD and *iv*NDFD were negatively correlated [43]. The *iv*DMD and *iv*OMD were significantly higher (*p* < 0.05) in the LB group than in the other groups in the middle to late ensiling stages (15 to 60 days), suggesting that the addition of *L. buchneri* may inhibit the proliferation of harmful microorganisms (e.g., moulds and clostridia), reduce the damage to the fibre structure (e.g., excessive accumulation of lignin), and lead to the retention of more digestible carbohydrates, thereby increasing OM digestibility [44].

Notably, the high abundance of *L. buchneri* in the LB group was directly correlated with AA accumulation and elevated aerobic stability. Bai et al. showed in a study of whole-plant corn silage that *L. buchneri* inhibits aerobic microorganisms through AA [33]. AA has inhibitory effects on clostridia and antioxidant properties, which increase the aerobic stability of silage and, in particular, plays a key role in suppressing the risk of secondary fermentation. The addition of *L. buchneri* significantly increased the aerobic stability of stringy-leafed pine brome for 51 h. This is consistent with our previous study [45] and is supported by a study by Nair et al., who showed a significant increase in aerobic stability with the addition of *L. buchneri* to corn silage [46].

As a key indicator, aerobic stability is related to the nutritional quality of ruminants and the subsequent utilisation value of feed. If silage can maintain high aerobic stability over time, it can significantly reduce the consumption of nutrients and the accumulation of mycotoxins in silage [47]. The aerobic stability of *S. perfoliatum* silage did not significantly increase with the addition of *L. plantarum*, which was attributed to the lack of yeast-inhibiting metabolites, such as AA, in the silage to effectively block the aerobic spoilage process when *L. plantarum* was used alone.

### 4.4. Effect of Different Types of LAB on the Bacterial Community Diversity, Composition and Succession of Silphium perfoliatum L. Silage

#### 4.4.1. Dynamics of Bacterial Community Diversity

In general, silages with high fermentation quality have low alpha diversity. In the *S. perfoliatum* silage, the alpha diversity was dynamic, as it increased and then decreased. *S. perfoliatum* may have a strong pH buffering capacity due to its high protein or mineral content, resulting in a slow decrease in pH at the initial stage. At this stage, LAB (especially the homofermentative LP group) failed to monopolise the resource completely, and the indigenous bacteria (e.g., Weissella) still proliferated, causing the diversity of the bacterial community to temporarily increase. Oxygen depletion and acidity deficiency led to the coexistence of Weissella with exogenous LAB, during which the bacterial diversity transiently increased. The increased activity of spoilage bacteria (e.g., Clostridium_B) and yeasts in the CK group led to an increase in microbial competition and the spontaneous proliferation of some flora (e.g., Lactiplantibacillus), which was reflected by the greater alpha diversity index in the CK group than in the LB group. AA produced by *L. buchneri* had a broad spectrum of inhibitory activities and selectively suppressed more bacterial groups (e.g., Weissella, Pantoea) during the middle fermentation period, resulting in significantly lower diversity in the LB group than the other two groups. At this stage, the bacterial community shifted towards *L. buchneri* dominance. The alpha diversity decreased in all groups at the late ensiling stage (45 to 60 days), reflecting the absolute dominance achieved by LAB (e.g., Lentilactobacillus and Lactiplantibacillus) through sustained acid production. With the depletion of soluble carbohydrates, only acid-tolerant bacteria and some fibre-degrading bacteria survived, and the community diversity decreased. *L. buchneri* in the LB group became the dominant genus at 45 days, and its allozyme fermentation products (such as AA) further suppressed other competing bacteria, leading to a continuous decrease in the diversity index. Although the alpha diversity fluctuated with ensiling time, the PCoA plots revealed no significant difference in the community structure over time. These findings suggest that the community structure stabilised in the middle and late stages of ensiling (30 to 60 days) and that the effect of ensiling time on bacterial diversity was not significant. According to the PCoA plot, it can be seen that the fresh samples were significantly separated from the treatment groups. This indicated that the ensiling process almost completely changed the original epiphytic bacterial community to form a fermentation community centred on LAB. The composition of the bacterial community in silage is related to the type of additive. The structure of the bacterial community in the LB group significantly differed from that in the control and LP groups, especially in the middle and late ensiling stages (30 to 60 days), which was closely related to the *L. buchneri*-mediated regulation of acetic acid metabolic pathways. These findings suggested that *L. buchneri* had a greater effect on the bacterial community of *S. perfoliatum* silage than *L. plantarum*. Xu et al. found that L. plantarum dominated in the early stage of corn silage [48]. Furthermore, the results of the study by Xu et al. are not in agreement with the results of the present study, as they reported that the effect of ensiling time on bacterial diversity was greater than that of additives in corn silage

#### 4.4.2. Changes in Dominant Bacterial Communities

This difference may be due to several factors, such as the type of silage, harvest time, and external environment. According to Yang et al., Pediococcus, Leuconostoc, and Weissella have the ability to produce LA and rapidly produce acid during the early stage of anaerobic fermentation (7–15 days) [49]. These bacteria are microorganisms that initiate the ensiling process and play crucial roles in the pre-ensiling period, effectively inhibiting the growth of moulds, yeasts, and spoilage bacteria, as well as preventing feed spoilage. These bacteria also work synergistically with LAB and yeasts to promote efficient fermentation and improve silage quality. The dominant genera in FM were Weissella_A and Pantoea_A, and the dominant species were *Weissella_A_338544 cibaria* and *Pantoea_A_680372 dispersa.* This result is in line with the characteristics of epiphytic flora on the surface of plants. Among them, Weissella is often involved in the metabolism of plant polysaccharides, while Pantoea is a parthenogenetic anaerobic bacterium that is always present during silage fermentation [50]. As harmful bacteria, Enterobacter and Escherichia species affect the quality of silage. Pathogenic bacteria of these two genera pose a threat to livestock health and cause various diseases [51]. The relative abundance of both genera was low in all the treatment groups, which indicated the excellent quality of the silage in this experiment. At 7 days of fermentation, the abundance of *Weissella_A_338544 cibaria* was significantly higher in the control group than in the LP and LB groups. It has been shown that the addition of *L. plantarum* and cellulase can reduce the relative abundance of Weissella and Leuconostoc [52]. Another study showed that the addition of 1 × 10^5^ and 1 × 10^7^ cfu/g *L. plantarum* reduced the relative abundance of Weissella in wolverine silage [53]. The abundance of Lentilactobacillus was significantly higher (*p* < 0.05) in the LB group than in the CK and LP groups at 7 days of fermentation. The abundance of *L. buchneri* was significantly higher (*p* < 0.05) in the LB group than in the CK and LP groups at 30 days of fermentation. These findings suggested that the *L. buchneri* inoculation accelerated the heterotrophic fermentation process. *L. buchneri*, through the simultaneous production of LA and AA, may inhibit *Weissella cibaria* and suppress pH decline. The abundance of *L. plantarum_A* in the LP group significantly increased after 30 days, reflecting the adaptation of homofermentative LAB in long-term acidified environments. *Clostridium tyrobutyricum* appeared in the CK group at 45 days. This finding suggested that the high pH of the uninoculated silage led to the proliferation of *Clostridium tyrobutyricum*, which may have triggered silage spoilage. Although *L. buchneri* became the dominant strain in the LB group at 45 days, its high AA production may have suppressed other LAB (e.g., *L. plantarum_A*), leading to a decrease in community diversity. These findings are consistent with the alpha diversity results.

#### 4.4.3. Comparative Analysis with Past Studies

Bai et al. explored the use of LAB additives on whole-plant corn silage and reported that the competitiveness of *L. plantarum* was greater in the pre-ensiling period but that the growth of *L. buchneri* was more vigorous in the later stages of ensiling [54]. However, in the present study, *L. plantarum* did not have an advantage in the pre-ensiling period. The reason for this difference may be that stringy pine balsam contains phenolic compounds (e.g., tannins and flavonoids), which are selectively inhibitory to some LAB (e.g., homofermentative bacteria). Heterofermentative bacteria may partially counteract the negative effects of antimicrobial substances in the raw material by generating AA with inhibitory effects, thereby preventing their survival [55]. LefSe analyses revealed key differential bacterial communities and their stage-specific functions during silage in the different treatment groups. The early enrichment of Pediococcus in the LB group, on the other hand, stemmed from its tolerance to the metabolite acetic acid of *L. buchneri*, which inhibited spoilage bacteria through synergistic acidification. At mid-silage (30 to 45 days), bacterial community differentiation intensified, with *L. plantarum_A* in the LP group monopolising the fermentation substrate to create a strongly acidified environment through homofermentation. Significant differences in the spoilage bacteria (e.g., Pantoea) in the CK group indicated the inadequacy of natural fermentation, with its aerobic metabolism leading to protein loss and the risk of warming. The continued dominance of Pediococcus in the LB group at the late silage stage (60 days) reflects its ability to stabilise symbiosis in an acetic acid environment, reinforcing aerobic stability through metabolic complementation.

### 4.5. Effect of Different Types of LAB on the Functional Predictions of Silphium perfoliatum L. Silage

Metabolism-related functions (50%) dominated silage microbial activities as seen from the KEGG pathway classification, and amino acid metabolism, carbohydrate me-tabolism, nucleotide metabolism, energy metabolism, and vitamin metabolism pathways were associated with changes in the fermentation characteristics and chemical composition of silage during silage fermentation.

PICRUSt2 functional prediction revealed that LAB types profoundly restructured the microbial metabolic network of *S. perfoliatum* silage. Amino acid metabolism, as a metabolic pathway that plays an important role in silage, is associated with protein hydrolysis induced by adverse microorganisms [56]. This is consistent with our expectations. At different ensiling periods, amino acid metabolism was significantly upregulated in the LB group. This is consistent with the trend that the CP content in the LB group was always higher than that in the LP and CK groups. Experiments have shown that the total abundance of LAB increased in groups with a higher abundance of carbohydrate metabolism pathways [21]. This is similar to the results of this experiment. Studies have shown that the total relative abundance of LAB in the microbial community affects the abundance of carbohydrate metabolism pathways. In this study, the relative abundance of energy metabolism in the LP and LB groups was higher throughout the entire silage stage.

This is similar to the results of Xu et al.’s study on whole-corn plants, which reported that energy metabolism in silage inoculated with lactic acid bacteria was upregulated during the middle stage of silage [57]. Nucleotides are primarily used for DNA synthesis and also provide energy for cells. Research by Kilstrup et al. indicates that bacterial utilisation of nucleotides influences most metabolic reactions [58]. During the 7-to-60-day silage process, the relative abundance of nucleotide metabolism in the LB group was higher than that in the LP group. This is contrary to the results of energy metabolism. This may be due to the higher total relative abundance of dominant LAB in the LB group and prolonged acid stress under anaerobic fermentation conditions. In addition, the sustained increase in cofactor and vitamin metabolism indicates that microorganisms maintain redox homeostasis through coenzyme synthesis, which has potential significance for extending the stability of silage feed during aerobic exposure.

## 5. Conclusions

Different types of LAB additives significantly affect the fermentation quality, chemical composition and in vitro digestibility of *S. perfoliatum* silage by modulating the microbial community structure and metabolic function. In this study, the effects of LAB additives on the fermentation quality, bacterial community, and predicted functions of *S. perfoliatum* silage were greater than those of the ensiling time. Inoculation with *L. plantarum* affected bacterial community succession in the pre-ensiling period, and inoculation with *L. buchneri* affected bacterial community succession in the middle and late ensiling periods. In addition, inoculation with *L. buchneri* was more effective for ensiling *S. perfoliatum* than inoculation with *L. plantarum* according to the predicted bacterial community changes and functions. Thus, *L. buchneri* should be selected as an additive for *S. perfoliatum* ensiling in practical production.

## Figures and Tables

**Table 1:** Chemical composition and buffering capacity of *Silphium perfoliatum* L.

Items ^‡^	Chemical Composition
Dry matter (% FW)	31.04
Organic matter (% DM)	85.07
Crude protein (% DM)	19.25
Neutral detergent fibre (% DM)	38.64
Acid detergent fibre (% DM)	29.72
Acid detergent lignin (% DM)	8.73
Water-soluble carbohydrate (% DM)	5.98
Buffering capacity (mEq kg^−1^ DM)	321.54

^‡^ FW, fresh weight; DM, dry matter.

**Table 2:** Fermentation quality of *Silphium perfoliatum* L. silage fermented for 7, 15, 30, 45, and 60 days.

Item	Day	Additives			Mean	SEM	Significance of Main Effects and Interactions		
		CK	LP	LB			D	A	D × A
pH value	7	4.87 ^B^	4.79 ^C^	4.96 ^C^	4.87	0.010	<0.001	0.003	<0.001
	15	4.82 ^B^	4.76 ^C^	4.88 ^C^	4.82				
	30	4.51 ^Aa^	4.54 ^Ba^	4.71 ^Bb^	4.59				
	45	4.47 ^Aa^	4.36 ^Aa^	4.80 ^BCb^	4.54				
	60	4.51 ^Ab^	4.31 ^Aa^	4.55 ^Ab^	4.45				
	avg	4.64	4.55	4.78					
NH_3_-N (%TN)	7	3.72 ^A^	3.81 ^AB^	4.05 ^B^	3.86	0.052	<0.001	<0.001	<0.001
	15	4.49 ^Ab^	3.31 ^Aa^	2.78 ^Aa^	3.53				
	30	3.15 ^Ab^	3.72 ^ABb^	2.15 ^Aa^	3.00				
	45	4.26 ^Ab^	4.77 ^Bb^	2.21 ^Aa^	3.75				
	60	7.86 ^Bb^	6.34 ^Cb^	2.58 ^Aa^	5.59				
	avg	4.70	4.39	2.75					
LA (%DM)	7	0.40	0.44 ^AB^	0.47 ^C^	0.44	0.010	<0.001	<0.001	<0.001
	15	0.36 ^b^	0.39 ^Bb^	0.26 ^Aa^	0.34				
	30	0.45 ^b^	0.46 ^ABb^	0.30 ^Aa^	0.40				
	45	0.52 ^b^	0.54 ^BCb^	0.34 ^ABa^	0.46				
	60	0.42 ^a^	0.58 ^Cb^	0.40 ^BCa^	0.46				
	avg	0.43	0.48	0.35					
AA (%DM)	7	0.13 ^B^	0.12	0.13 ^AB^	0.13				
	15	0.09 ^A^	0.11	0.06 ^A^	0.09				
	30	0.07 ^A^	0.07	0.08 ^AB^	0.07	0.003	<0.001	0.392	0.005
	45	0.07 ^A^	0.08	0.08 ^AB^	0.08				
	60	0.10 ^Aa^	0.11 ^a^	0.14 ^Bb^	0.12				
	avg	0.09	0.10	0.10					

^a,b^ Different lowercase letters indicate significant (*p* < 0.05) differences between means of different additive treatments for the same number of days of silage. ^A–C^ Different capital letters indicate significant (*p* < 0.05) differences between means of different silage days for the same additive treatment. SEM, standard error of the mean; D, Days of silage; A, additive; CK, control group (no additive group); LP, *Lactiplantibacillus plantarum* added group; LB, *Lentilactobacillus buchneri* added group; NH_3_-N, ammonia nitrogen; TN, total nitrogen; LA, lactic acid; AA, acetic acid; DM, dry matter.

**Table 3:** Chemical composition of *Silphium perfoliatum* L. silage fermented for 7, 15, 30, 45, and 60 days.

Item	Day	Additives			Mean	SEM	Significance of Main Effects and Interactions		
		CK	LP	LB			D	A	D × A
DM (%FW)	7	27.30	27.70 ^C^	27.06 ^A^	27.35	0.205	0.002	<0.001	<0.001
	15	25.66 ^a^	27.38 ^Ca^	31.83 ^Bb^	28.29				
	30	27.18 ^a^	24.54 ^ABa^	32.25 ^Bb^	27.99				
	45	25.45	23.47 ^A^	26.45 ^A^	25.12				
	60	25.40	25.94 ^BC^	27.87 ^A^	26.41				
	avg	26.20	25.81	29.09					
OM (%DM)	7	84.60 ^B^	84.85	84.58	84.68	0.017	<0.001	0.802	<0.001
	15	84.85 ^Cb^	84.96 ^b^	84.49 ^a^	84.76				
	30	84.35 ^A^	84.62	84.59	84.52				
	45	84.51 ^ABa^	84.57 ^ab^	84.72 ^b^	84.60				
	60	85.02 ^Db^	84.77 ^a^	84.55 ^a^	84.78				
	avg	84.67	84.75	84.59					
CP (%DM)	7	17.36 ^B^	16.90 ^B^	17.12 ^A^	17.13	0.038	<0.001	<0.001	<0.001
	15	16.63 ^Ba^	18.00 ^Cb^	18.91 ^Bc^	17.85				
	30	18.80 ^Cb^	16.89 ^Ba^	19.61 ^Cc^	18.43				
	45	15.53 ^Aa^	15.87 ^Aa^	19.29 ^BCb^	16.90				
	60	15.21 ^Aa^	16.18 ^ABb^	17.58 ^Ac^	16.32				
	avg	16.71	16.77	18.50					
NDF (%DM)	7	37.73 ^BC^	39.38 ^C^	37.28 ^C^	38.13	0.132	<0.001	<0.001	<0.001
	15	35.85 ^ABb^	36.43 ^Bb^	32.45 ^ABa^	34.91				
	30	33.44 ^A^	32.74 ^A^	35.22 ^BC^	33.80				
	45	36.50 ^Bc^	34.48 ^Ab^	31.03 ^Aa^	34.00				
	60	39.68 ^Cb^	38.98 ^Cb^	34.69 ^BCa^	37.78				
	avg	36.68	36.40	34.13					
ADF (%DM)	7	26.16 ^A^	26.82 ^AB^	26.29 ^C^	26.42	0.115	<0.001	<0.001	<0.001
	15	28.10 ^Bb^	25.50 ^Aa^	25.28 ^BCa^	26.30				
	30	24.93 ^Aa^	27.85 ^BCb^	24.26 ^ABa^	25.68				
	45	29.66 ^Cb^	29.04 ^Cb^	23.03 ^Aa^	27.24				
	60	29.27 ^BCb^	28.14 ^BCb^	25.90 ^BCa^	27.77				
	avg	27.62	27.47	24.95					
ADL (%DM)	7	8.39 ^C^	7.86 ^C^	8.59 ^D^	8.28	0.069	<0.001	<0.001	<0.001
	15	3.25 ^Aa^	3.86 ^ABa^	4.91 ^BCb^	4.01				
	30	4.74 ^Bab^	3.36 ^Aa^	5.62 ^Cb^	4.57				
	45	5.17 ^B^	4.92 ^B^	3.87 ^AB^	4.65				
	60	4.12 ^AB^	4.29 ^AB^	3.45 ^A^	3.95				
	avg	5.13	4.86	5.29					

^a–c^ Different lowercase letters indicate significant (*p* < 0.05) differences between means of different additive treatments for the same number of days of silage. ^A–D^ Different capital letters indicate significant (*p* < 0.05) differences between means of different silage days for the same additive treatment. SEM, standard error of the mean; D, Days of silage; A, additive; CK, control group (no additive group); LP, *Lactiplantibacillus plantarum* added group; LB, *Lentilactobacillus buchneri* added group; DM, dry matter; FW, fresh weight; OM, organic matter; CP, crude protein; NDF, neutral detergent fibre; ADF, acid detergent fibre; ADL, acid detergent lignin.

**Table 4:** In vitro digestibility of *Silphium perfoliatum L.* silage fermented for 7, 15, 30, 45 and 60 days.

Item	Day	Additives			Mean	SEM	Significance of Main Effects and Interactions		
		CK	LP	LB			D	A	D × A
*iv*DMD (%DM)	7	66.15 ^AB^	65.19 ^A^	66.41 ^A^	65.91	0.076	<0.001	<0.001	<0.001
	15	67.24 ^BCa^	66.90 ^Ba^	69.21 ^BCb^	67.78				
	30	68.64 ^C^	69.04 ^C^	67.61 ^AB^	68.43				
	45	66.86 ^Ba^	68.03 ^Cb^	70.03 ^Cc^	68.31				
	60	65.01 ^Aa^	65.42 ^Aa^	67.91 ^ABb^	66.12				
	avg	66.78	66.92	67.79					
*iv*OMD (%DM)	7	69.88 ^AB^	68.93 ^A^	70.13 ^A^	69.65	0.075	<0.001	<0.001	<0.001
	15	70.95 ^BCa^	70.62 ^Ba^	72.90 ^BCb^	71.49				
	30	72.34 ^C^	72.74 ^C^	71.32 ^AB^	72.13				
	45	70.58 ^Ba^	71.74 ^Cb^	73.71 ^Cc^	72.01				
	60	68.76 ^Aa^	69.16 ^Aa^	71.62 ^ABb^	69.85				
	avg	70.50	70.64	71.49					
*iv*CPD (%DM)	7	57.71 ^Db^	56.33 ^Aa^	56.92 ^Aa^	56.99	0.043	<0.001	<0.001	<0.001
	15	56.75 ^Ca^	57.86 ^Bb^	59.40 ^Bc^	58.00				
	30	59.12 ^Eb^	57.55 ^Ba^	59.51 ^B^	58.72				
	45	55.65 ^Ba^	56.32 ^Aa^	60.89 ^Cb^	57.62				
	60	54.77 ^Aa^	55.77 ^Ab^	57.80 ^Ac^	56.11				
	avg	56.80	56.77	59.11					
*iv*NDFD (%DM)	7	36.50 ^BC^	37.85 ^C^	36.12 ^C^	36.82	0.112	<0.001	<0.001	<0.001
	15	34.84 ^ABb^	35.32 ^Bb^	32.03 ^ABa^	34.06				
	30	32.90 ^A^	32.25 ^A^	34.36 ^BC^	33.17				
	45	35.47 ^Bc^	33.74 ^Ab^	30.77 ^Aa^	33.33				
	60	38.07 ^Cb^	37.52 ^Cb^	33.92 ^BCa^	36.50				
	avg	35.58	35.34	33.44					
*iv*GED (%DM)	7	69.74 ^AB^	68.81 ^A^	69.99 ^A^	69.52	0.074	<0.001	<0.001	<0.001
	15	70.80 ^BCa^	70.47 ^Ba^	72.70 ^BCb^	71.32				
	30	72.15 ^C^	72.54 ^C^	71.15 ^AB^	71.95				
	45	70.43 ^Ba^	71.56 ^Cb^	73.50 ^Cc^	71.83				
	60	68.65 ^Aa^	69.04 ^Aa^	71.45 ^ABb^	69.71				
	avg	70.35	70.48	71.32					

^a–c^ Different lowercase letters indicate significant (*p* < 0.05) differences between means of different additive treatments for the same number of days of silage. ^A–E^ Different capital letters indicate significant (*p* < 0.05) differences between means of different silage days for the same additive treatment. SEM, standard error of the mean; D, Days of silage; A, additive; CK, control group (no additive group); LP, *Lactiplantibacillus plantarum* added group; LB, *Lentilactobacillus buchneri* added group; DM, dry matter; *iv*DMD, in vitro dry matter digestibility; *iv*OMD, in vitro organic matter digestibility; *iv*CPD, in vitro crude protein digestibility; *iv*NDFD, in vitro neutral detergent fibre digestibility; *iv*GED in vitro gross energy digestibility.

## Data Availability

The data supporting this study are included in the article; further inquiries can be directed at the corresponding authors.
